# Improving Fire Performances of PEAL: More Second-Life Options for Recycled Tetra Pak^®^

**DOI:** 10.3390/polym12102357

**Published:** 2020-10-14

**Authors:** Fulvia Cravero, Alberto Frache

**Affiliations:** Department of Applied Science and Technology, Politecnico di Torino, Viale Teresa Michel 5, 15121 Alessandria, Italy; fulvia.cravero@polito.it

**Keywords:** Tetra Pak^®^, PEAL, recycling, cone calorimeter, intumescent system, magnesium hydroxide

## Abstract

The purpose of this work was to evaluate and improve the flammability and combustion behavior of the polyethylene-based material obtained from the recycling of Tetra Pak^®^ (PEAL) to widen its use to applications where these properties are required. Firstly, its thermal stability was investigated with thermogravimetric analysis, resulting in an enhancement in the main degradation step temperature (from 385 °C to 421 °C) due to the presence of the aluminum-flakes. Then, to improve the poor flammability (HB in UL-94 test) and combustion behavior (Fire Performance Index of 0.07) of the raw material, two flame retardant approaches were tested: an intumescent system made of ammonium polyphosphate and pentaerythritol, and magnesium hydroxide. In addition, the effectiveness of polyethylene as a charring agent was evaluated. Characterization was made with UL-94, cone calorimeter, and morphologic analysis. For all the materials tested, the temperature of the main weight loss step increased and the flammability rating improved (V2 for intumescent and V0 for magnesium hydroxide reached). Moreover, fire hazard decreased (Fire Performance Index of 0.15 and 0.55; Flame Retardancy Index of 2.6 and 10.0). Referring to the morphology, full compatibility was found in the PEAL–magnesium hydroxide compound, while PEAL-intumescent appeared as a heterogeneous system.

## 1. Introduction

In the last decades, more attention has been paid to the problem of plastic waste discarded into the environment or landfilled [1]. In particular, an important volume is represented by multilayer packaging used for food purposes, also known as Tetra Pak^®^ packaging. This is a system made by layers of polyethylene (PE), aluminum and paperboard that can vary in relative concentrations and layer arrangement, depending on the kind of food contained (e.g., prepared food, beverage, cheese, whey) and on the preservation technique chosen (long-life, frozen or fresh) [2,3]. The main problem with this kind of packaging is that individually, the three materials are recyclable, but in multilayer form, they stick together (especially PE and aluminum) and are so difficult to separate that, in most cases, require to be processed together [4].

In particular, in Italy, multilayer packaging has been treated as unsorted waste starting from their introduction (about 1951 [5]) up to 2003, because the production volume was too low to introduce a separate waste collection [6]. The solution in use nowadays is to collect Tetra Pak^®^ wastes with paper-ones and process them in a paper factory to remove the high quantity of paperboard and recycle it as paper. The by-product, constituted of polymers (such as PE, polypropylene but also polyamides and polyesters), aluminum and other impurities, can undergo different destinies [7]. The older one is to landfill. This has been then integrated with energy recovery, thanks to the calorific value of polymers. In this way, the impact on the environment is reduced but is completely lost the added-value acquired by the material during the production and forming processes. In more recent years, a third approach is spreading. It consists in the elimination of the impurities and the foreign polymers from the PE-aluminum mix, giving an added-value product that can be injected, extruded, blended and compounded like a normal polymer, and further recycled. This material is a product, in Italy, by Ecoplasteam S.p.a [8] with the trade name EcoAllene^®^ AA00 BASE (PEAL in this work).

Despite the advantages in giving a second life to the by-product, application fields are still largely under study. Active production lines and potentials refer to packaging, toys, accessories, furnishing and home decor, gardening, and office supplies [9]. The researchers have focused primarily on the characterization of the material such as thermal analysis (Thermogravimetric Analysis - TGA, Differential Scanning Calorimetry - DSC) [10,11], Fourier Transform Infrared Spectroscopy (FTIR) [11], rheological characteristics [11], and Scanning Electron Microscopy (SEM) analysis [11]. Also, a thermal conductivity and heat capacity (Modulated Differential Scanning Calorimetry - MDSC) study is available [12]. Moreover, interest was shown for mechanical properties [11] and the effects on these natural fibers or powders additivation [10,13,14].

Flammability evaluation have been done by Xu et al. [15] on the recycled Tetra Pak^®^ still containing paperboard and in the presence of 26 %*w* of High Density-PE (HDPE). In the study, V2 classification has been achieved with the addition of 20 %*w* of ammonium polyphosphate (APP), while with 40 %*w* of APP, V0 classification was obtained [15]. On the other hand, no paper on the PEAL alone has been found. This is of particular interest considering applications like electrical components (such as electrical ducts and pipes) and public transport (seats, overhead bin), where flame retardant (FR) characteristics for the materials used are needed. In addition, depending on the specific application and component a different level of flame retardancy is required. These applications are of particular interest because nowadays, PE is largely used for the above components, so replacing with the recycled material would save a huge quantity of the neat PE. In addition, both for transports and electric components, the service life can be even of 10–15 years. This involves that the by-product would not be considered as waste for an additional decade or more, if compared with the first service life as food packaging.

For the above reasons, in this work, an innovative approach to PEAL, involving FR additives, is proposed. In the absence of referenced literature for the recycled material, studies on PE have been analyzed [16,17,18,19,20,21,22,23,24]. In particular, to maintain the cost-effectiveness of PEAL, the simplest formulations have been selected among the others.

The first class of FR investigated was the intumescent (IFR) one. Even if the effectiveness of this kind of additive is, in general, low due to the poor compatibility with the matrix [16], Han et al. [17] as well as Khanal et al. [18], tried the success of PE itself as a charring agent, in the presence of APP. Also, the benefit of a proper charring agent has been deepened by Lu et al. [16], Han et al. [17] and Wu et al. [19], with the introduction of Pentaerythritol (PER). In particular, the concentration of IFR reported in the previous documents is always between 30 %*w* and 45 %*w*.

The second approach analyzed involved inorganic additives. Due to the process temperature of 180 °C, just the papers reporting magnesium hydroxide (MH) have been taken into account. Specifically, with the studies of Lenza et al. [20], Wang et al. [21], Liu [22], Zhang et al. [23] and, Hornsby et al. [24]. The previous studies are in accordance when reporting the best FR performances with a concentration of additive between 50 %*w* and 70 %*w*.

The present work is, therefore, divided into two parts. Due to the lack of information referring to the flammability and thermal stability of PEAL, in the first part an in-depth characterization of the material has been done. In particular, has been evaluated the thermal stability in inert and oxidative atmosphere and flammability of the material.

The second part focuses on the characterization and comparison of the FR compounds with PEAL performances via TGA, UL-94 and cone calorimeter. The latter, in order to analyze also the combustion behavior. In addition, SEM has been analyzed the shape, dimension, distribution and compatibility of aluminum and additives with the matrix.

## 2. Materials and Methods

### 2.1. Materials

PEAL is a polyolefin-based material composed on average by 85 %*w* Low Density-PE (LDPE) (polypropylene and HDPE- traces were present in the material) and by about 15 %*w* of aluminum. In particular, the material has been subjected to a filtration process with a light of 400 µm by EcoPlasteam S.p.a, in order to reduce the concentration and size of aluminum contained. Neat LDPE (Borealis CA7230) is supplied in pellets by EcoPlasteam S.p.a. (Alessandria, Italy). APP in powder form (type I, Budit series) has been produced by Budenheim Iberica S.l.u. (Saragoza, Spain). PER in powder form was supplied by SIGMA–ALDRICH Chemie GmbH (purity level > 98%, Munich, Germany). MH in powder form (particle size 1–5micron) has been provided by De Grandi s.r.l. (Pavia, Italy).

IFR was prepared with a 3:1 weight ratio between APP and PER manually mixed with a spoon for 60 s at room temperature.

### 2.2. Preparation of the Compounds

FR compounds were prepared using a PlastiCorder Brabender model W50E (Duisburg, Germany) with counter-rotating screws in which PEAL and FR additives were added. The processing was performed in air at 180 °C with a screw speed of 30 rpm during loading and of 60 rpm for 3 min during maintenance. Formulations obtained are reported in Table 1.

After compounding, the materials have been ground with a single step in Piovan S25 rotor granulator (Venice, Italy). Furthermore, the samples for UL-94, cone calorimeter and SEM tests have been obtained via compression moulding with Collin P 200 T press (Maitenbeth, Germany). In all cases, the processing has been performed in air at 180 °C with a plastification time of 3 min under a growing pressure (maximum of 50 bar). Fifteen degassing cycles have followed with a pressure of 50 bar. Maintenance lasted 2 min under a pressure of 100 bar. The extraction of the material was done at a temperature of 75–85 °C.

### 2.3. Characterizations

#### 2.3.1. TGA

TGA has been used to evaluate the thermal (nitrogen atmosphere) and thermoxidative (air atmosphere) resistance of PEAL and FR compounds. Three parameters have been taken particularly into account: the temperature of the beginning of degradation (T_2%_), assumed to be the temperature correspondent to a residual weight of 98%; the reference temperature for the main weight-loss step (T_max_), identified as the peak of the derivative of the weight on the temperature, and the amount of final residue at 800 °C. In general, the higher and grater the temperatures and the weight, the higher the stability of the material.

TGA analysis were performed using a Discovery TA Instrument (New Castle, DE, USA) in nitrogen or air on single-grain samples of 10–12 mg average weight, contained in alumina pans. Data have been recorded between 50 °C and 800 °C, with a heating ramp of 10 °C/min.

#### 2.3.2. UL-94

UL-94 analysis was used to evaluate flammability according to ASTM D 635 [25] and ASTM D 3801 [26] for horizontal and vertical setting, respectively. Classification is, in ascending order of flame retardancy, HB if the material respects the parameters prescribed for the horizontal configuration [25] while V2, V1 and V0 refers to the attribution of the flame retardancy level in accordance with the vertical setting [26]. V0 has to be intended as the best and ideal performance expected.

In particular, for the horizontal configuration measurement have been monitored the speed of combustion, the spreading of the flames, the quantity and quality of the dripping. On the other hand, in vertical configuration time of combustion, the quantity and quality of the dripping have been monitored. Standard deviation has been reported for the results.

Three samples of 80 × 13 × 2 mm for each material have been conditioned in climatic chamber at 23 °C and 50 %rh for 48 h. The Bunsen burner flame was 20 mm in height.

#### 2.3.3. Cone Calorimeter

Combustion behavior has been evaluated with Fire Testing Technology Limited (*FTT*) cone calorimeter (West Sussex, UK), according to ISO 5660 [27,28]. In particular, have been monitored: Time to Ignition (*TTI*), Heat Release Rate (or *HRR*), Total Heat Release (*THR*), Total Smoke Release (or *TSR*—related to smokes opacity) and final residue. In particular, for *HRR* both the peak value (Peak HRR) and Time of Peak HRR have been monitored.

In addition, two important parameters have been calculated. The first one is Fire Performance Index (*FPI*) [29], which is inversely proportional to the hazard of the fire and refers specifically to each material. *FPI* can be calculated in accordance with Equation (1).

(1) $$FPI=\frac{TTI}{Peak\;HRR}\;\left[\frac{sm^{2}}{kW}\right]$$

The second is Flame Retardancy Index (*FRI*) [30]. This one, is a dimensionless parameter introduced by Vahabi et al. [30] for thermoplastics with the purpose of compare the fire retardancy characteristics of the starting material with the ones of the FR-composites, when tested with cone calorimeter. *FRI* can be calculated in accordance with Equation (2).

(2) $$FRI=\frac{{\left[THR*\left(\frac{Peak\;HRR}{TTI}\right)\right]}_{Neat\;polymer}}{{\left[THR*\left(\frac{Peak\;HRR}{TTI}\right)\right]}_{Composite}}$$

Flame retardancy characteristics of the composites are defined as poor (*FRI* < 1), good (1 < *FRI* < 10^1^) or excellent (10^1^ < *FRI* < 10^2^) [30].

Moreover, for all the previous parameters, the standard deviation and the percentage deviation from PEAL has been calculated.

Three samples of 50 × 50 × 3 mm have been tested for each material, after being conditioned in climatic chamber at 23 °C and 50 %rh for 48 h. The heat flux was set at 35 kW/m^2^ with a distance between the heating source and the sample of 25 mm, thus resulting in a superficial temperature of 681 °C.

#### 2.3.4. SEM

Distribution, dimension and shape of the flakes and FR additives have been evaluated with SEM along with the compatibility with the matrix. Morphology of the compound has been observed using an EVO 15 Zeiss Scanning Electron Microscope (beam voltage: 20 kV; backscattered electrons, Oberkochen, Germany). Observation surfaces have been obtained through fracturing in liquid nitrogen and coating with a gold layer of samples of dimension 80 × 13 × 2 mm.

## 3. Results and Discussion

### 3.1. PEAL

#### 3.1.1. TGA

TGA analysis of PEAL has been carried out both in nitrogen and air. Moreover, the material has been compared to LDPE. In Table 2, the main results have been listed.

**Figure 1:**
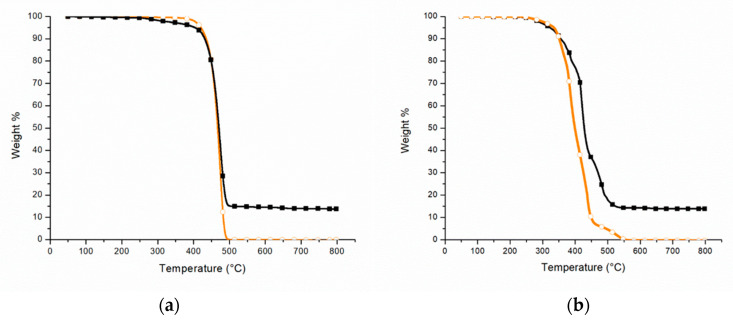
TGA in (**a**) nitrogen and (**b**) air of PEAL (black square) and LDPE (orange circle).

In an inert atmosphere (Figure 1a), the T_2%_ of PEAL (black square) is about 312 °C, while for LDPE (orange circle) is approximately 402 °C. The difference between the two values may be due to the origin of the PEAL. In fact, this recycling material contains impurities that can promote the degradation process. On the other hand, it does not seem to affect the temperature of the main weight-loss step which reflects that of LDPE, at approximately 474 °C, in accordance with the results of Hidalgo et al. [10] and Munoz et al. [13] (about 470 °C for both). The residue at 800 °C reflects the presence of aluminum, of about 14 %*w*. The result is in accordance with what was reported by Lopes et al. [11] (15 %*w*) but not with those stated by Hidalgo et al. [10] and Munoz et al. [13] (25 %*w*). This difference may be due to the production process patented by Ecoplasteam S.p.a. [31], which includes a filter for the size and content reduction of aluminum content in PEAL.

Figure 1b reports the thermograms of PEAL (black square) and LDPE (orange circle) in oxidative atmosphere. The result obtained for the recycled material is in accordance with Lopes et al. [11] and, in particular, in both cases, three weight-loss steps can be distinguished. During the first one at 386 °C, the degradation starting from the chains defects is occurring. The second step at 421 °C, is due to the random cracking of the chains for the proceeding of dehydrogenative oxidation, resulting in the formation of low C-content products that are stable up to higher temperatures. In fact, these residues (char) degrade due to oxidation during the third weight-loss step, which can be distinguished at 484 °C.

It worth noting that even if in accordance with Lopes et al., some differences can be found both in temperature ranges and weight-losses. This phenomenon can be explained with the different service life and processes to which the two materials were subjected, which affect the chain structure and so the proceeding of dehydrogenative oxidation.

In addition, the comparison in the TGA of PEAL and LDPE (Figure 1b) exhibits a comparable T_2%_ (283 °C for PEAL and 294 °C for LDPE) while they differ in the T_max_ (421 °C for PEAL and 385 °C for LDPE), with an increase of about 35 °C for PEAL. As reported by Lopes et al. [11], the higher thermoxidative stability of the aluminum-containing material may be due to the barrier effect of the metal particles on oxygen diffusion in the polymer matrix.

The residue at 800 °C for PEAL is not affected by the atmosphere of the test (Table 2).

#### 3.1.2. SEM

**Figure 2:**
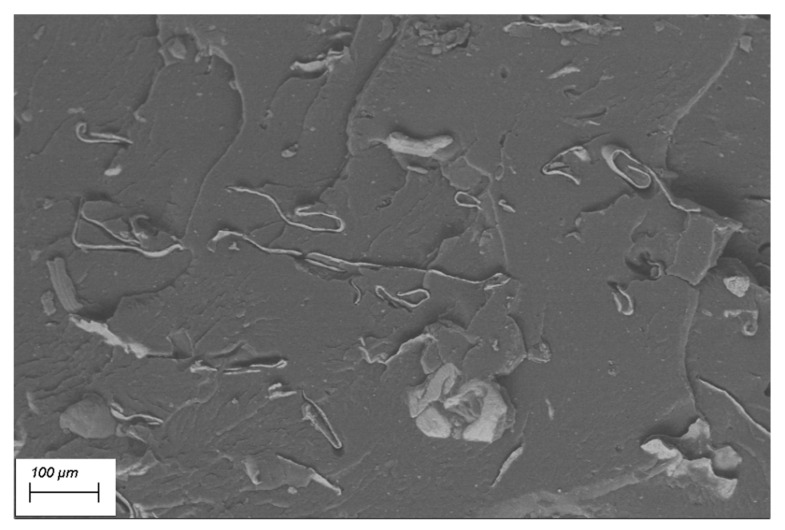
SEM image 250× of compression-moulded PEAL.

The SEM image at 250× (Figure 2) shows the section of a PEAL sample obtained via compression moulding. The aluminum is homogeneously dispersed in the matrix in platelet-like shape. The main dimension is, on average of a few hundreds of micrometres.

Despite the good dispersion and the incorporation of the aluminum, there is a poor compatibility with the polymer as can be seen by the voids at the interface between the metal and the matrix.

### 3.2. FR PEAL

#### 3.2.1. TGA

The TGA analysis of the FR compounds has been tested in oxidative atmosphere. The T_2%_, T_max_ and the residues at 800 °C are listed in Table 3.

**Figure 3:**
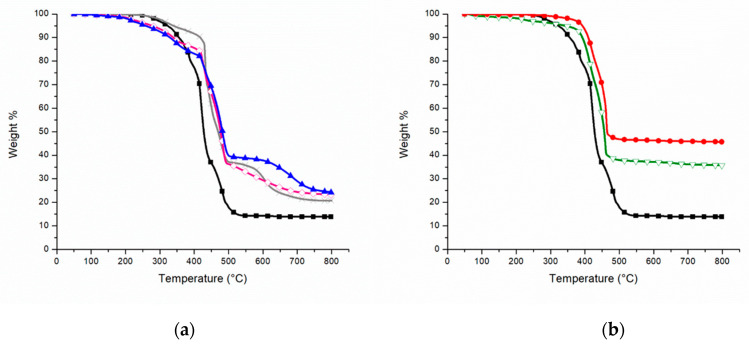
TGA in air of (**a**) PEAL (black square), PEALAPP 30 (grey cross), PEALIFR3031 (pink rhombus), PEALIFR4031 (blue triangle); (**b**) PEAL (black square), PEAL50MH (green triangle), PEAL60MH (red circle).

In Figure 3a, the behavior of the IFR formulations in comparison with PEAL (black square) is shown. If for PEALAPP30 (grey cross) T_2%_ is higher than PEAL (295 °C instead of 283 °C), for both PEALIFR3031 (pink rhombus) and PEALIFR4031 (blue triangle) degradation begins at lower temperature, around 212 °C. On the other hand, all the IFR compounds show three weight-loss steps similar to each other. With the first one, there is a reduction of about 10–20 %*w* and the reference temperatures are approximately 330–340 °C. The second weight-reduction step (T_max_) at about 480 °C affects approximately 40% in weight. Lastly, the third step lowers of about 15 %*w* and the reference temperatures are approximately 550 °C and 600 °C. Also, the final residue at 800 °C does not differ so much among the three compounds, around 23 %*w*.

As explained by Camino et al. [32] the decrease in T_2%_ is functional for the formation of the intumescent barrier and is the result of the reactions for the development of gasses (water and ammonia). This can be appreciated by the temperature of the main weight-loss step, which is about 50 °C higher than PEAL one (Figure 3a). Moreover, up to 400 °C the weight of the compounds (on average −12 %*w*) decreases less quickly than weight of PEAL (−23 %*w*). In addition, the final residue has increased by about 6–10%.

In the end, all the intumescent formulation tested seem to be more stable to thermooxidation if compared to PEAL as received.

Figure 3b shows the comparison between PEAL (black square) and the same material compounded with MH at 50 %*w* (green triangle) and 60 %*w* (red circle) respectively. The materials show two distinct weight-loss steps at comparable temperatures (about 417 °C and 467 °C for PEAL50MH; 420 °C and 462 °C for PEAL60MH).

Taking into consideration that there is no evidence in literature of TGA in air of PEAL flame retarded with MH, the results have been compared with the ones referring to FR PE. In particular, to the study of Lenza et al. [20] on HDPE compounded with 10–55 %*w* MH. The temperature of the beginning of degradation (T_1%_) was in all cases between 350 °C and 360 °C. Also, two main steps of degradation have been reported (the first between 382 °C and 401 °C, the second between 477 °C and 481 °C). These two steps have been explained as follows, the first one corresponds to the dehydration of MH while the second is due to the degradation of the matrix. Considering the variation in temperature due to the different matrix, the weight-loss steps of both PEAL50MH and PEAL60MH are in accordance with the explanation of Lenza et al.

The increase of the thermoxidative stability of the two main degradation steps in both materials and the number of final residues (Table 3), are important to underline in the analysis of these materials.

#### 3.2.2. UL-94

Once we have seen that all the FR formulations show an increasing in the thermal stability, such have been tested for flammability. This was necessary because no other study relative to flammability of PEAL has been found. In general, the materials have been tested in horizontal and later on in vertical configuration.

**Figure 4:**
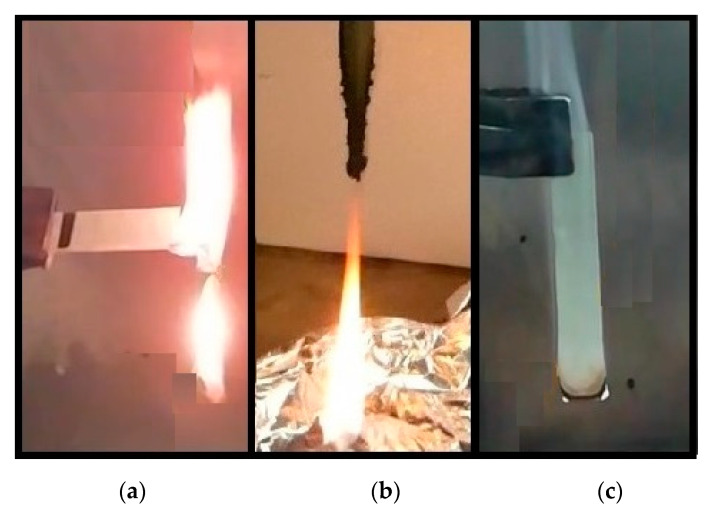
(**a**) PEAL as such in the horizontal test; (**b**) PEALIFR4031 in vertical test; (**c**) PEAL60MH in vertical test.

Figure 4a shows the picture of horizontal UL-94 test for PEAL. The material undergoes complete combustion and ignites few seconds before LDPE. It drips discrete portions of flamed material during combustion while for the pure LDPE, there is a continuous flamed filament [33]. In addition, speed of combustion of PEAL is about 39 (±3) mm/min, which means the material can be classified as HB [25]. Due to the poor flammability resistance PEAL has not been tested in a vertical position.

PEALAPP30 in horizontal configuration burns completely with flamed drips, similar to PEAL. In addition, the material does not show char formations. However, the speed of combustion is on average 26 (±3) mm/min, so lower than PEAL and comparable with HB classification [25]. Despite the reduction observed, the material shows poor flammability resistance and no intumescent structures, so it has not been tested in a vertical position.

For PEALIFR3031 in horizontal testing, flames extinguish before the first mark, so it can be classified HB [25]. At the same time, it has been appreciated char leftover on the burned portion of the samples. However, it has to be taken into account that the extinction is due to the dripping of the flamed portion of material.

In the vertical configuration, the material is not classifiable because flames always reach the clamps [26]. Moreover, the char brings out the poor interaction between gasses and cellular framework, which does not appear in the typical intumescent “bubble structure”. On the other hand, it is worth noting that in all the samples, flames extinguish after the first application due to the dripping of the flaming material. In addition, in two cases, the leftover portion of the sample is still structurally consistent and in one of them, the flame is extinguished via dripping even after the second application. The average time of combustion after the first and the second application is 17 (±1) s and 14 (±6) s respectively.

PEALIFR4031 in horizontal configuration has shown the best behaviour among the IFR compounds tested. In particular, even during the application of the external flame it has been appreciable the formation of the intumescent bubble structure and the extinction of the flames occurred before the first mark without any dripping. The material is classified HB [2,5] and it is also tested in VB configuration.

As can be seen in Figure 4b, the sample tested extinguished after both the applications with average times of combustion of 2 (±1) s and 11 (±3) s, respectively. In all cases, the extinction after the second application is due to dripping material, so PEALIFR4031 is classifiable V2 [26]. Also, all the samples showed the intumescent bubble structure starting from the first application, even in portion of the material not directly in contact with the flames. This structure evolved during the combustion from a large small-size number to few larger bubbles distributed along the contours of the samples.

These results disagree with the ones of Lu et al. [16]. In fact, for a material consisting in 40 %*w* IFR (APP:PER = 3:1) and 60 %*w* matrix (HDPE:EVA = 75 %*w*:25 %*w* with 5 %*w* of organically modified montmorillonite—OMT) has been report a V-0 classification. The great disparity with the tested material may be explained with the substantial difference in the composition. Firstly, the matrix was mainly made of neat HDPE instead of mixed and recycled LDPE/HDPE/PP. In addition, the formulation contained EVA, well known as a compatibilizer, and especially OMT, used as anti-dripping purposes in FR applications. In conclusion, PEALIFR4031 could be considered as a selected material considering the simplicity of the formulation and the recycling provenance.

Moving on the MH-FR materials, in all the samples of PEAL50MH tested in horizontal configuration, flames extinguished before the first mark. The material has been classified HB [25]. However, in vertical configuration the average burning time after the first application is 54 (±2) s. In addition, the extinction of the flames is due to the dripping of the flamed material. Because of that, PEAL50MH is nonclassifiable in vertical conditions [26]. This result is different from the one obtained by Lenza et al. [20] with a HDPE + 50 %*w* MH, in fact in that case, V0 classification at UL-94 with no dripping was achieved. Once more, the difference can be attributable to the lower quality of the polymer matrix.

Lastly, PEAL60MH has been tested in the vertical configuration because already in the case of the compound with a lower concentration of FR flames extinguished before the first mark in horizontal configuration.

All the samples extinguished the flames after both the applications with average times of combustion of 2 (±2) s and 2 (±2) s respectively. Moreover, as can be seen in Figure 4c, no dripping has been observed. PEAL60MH has been classified V0 [26].

It worth noting that after the flame contact, all the samples showed swelling in the lower part. This swollen material was subjected to glowing.

In conclusion, for further investigations PEALIFR4031 has been selected as the more performing intumescent material while PEAL60MH has been chosen between the MH flame-retarded ones.

#### 3.2.3. Cone Calorimeter

Combustion behaviour of PEALIFR4031 and PEAL60MH has been examined via cone calorimeter and compared with PEAL. In Table 4 the main results have been listed.

**Figure 5:**
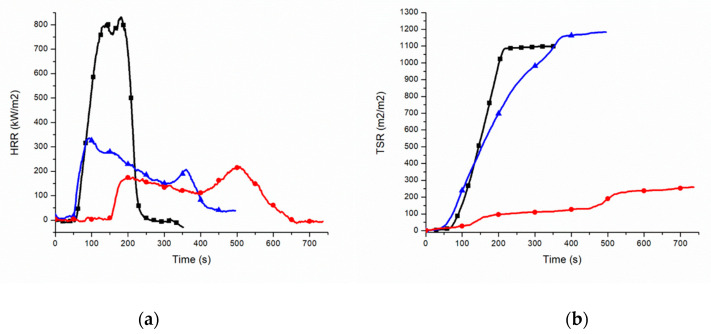
(**a**) HRR PEAL (black square), PEALIFR4031 (blue triangle), PEAL60MH (red circle); (**b**) TSR PEAL (black square), PEALIFR4031 (blue triangle), PEAL60MH (red circle).

In Figure 5a the curves of HRR are shown. The TTI is comparable for PEAL and PEALIFR4031, while for PEAL60MH the value is three times higher. In addition, the time and intensity of the Peak HRR are different among the materials. A different attitude can be appreciated also considering the shape of the curves. For both the FR material the value of HRR is more constant if compared with PEAL. In fact, the FPI of the three materials shows that for both the FR material the danger of the combustion decreases and that the hydroxide-containing one gives the more controlled option in terms of HRR developed. This is in accordance with FRI, the values of which define as good and excellent the flame retardancy features of PEALIFR4031 and PEAL60MH, respectively (Table 4).

Investigating the literature, few reports have been found analysing the combustion behaviour of PE-based intumescent compounds. In particular, Lu et al. [16] compared the mix HDPE:EVA = 75 %*w*:25 %*w* containing 5 %*w* OMT with the same material flame-retarded via 40 %*w* IFR mix (APP:PER = 3:1). Even if both the reported and the tested materials contained PE and 40 %*w* IFR 3:1, these are different materials due to the kind and provenance of the matrix and the presence of OMT. At the same time, no other evidence of more similar materials has been found in literature, so these data have been used as a general trend comparison.

Referring to the time of both ignition and peak of HRR, Lu et al. report a delay of 9 s and 279 s respectively, while in the present study, both of the times preceded PEAL as such. On the other hand, in both cases there is a reduction in the HRR peak intensity, even if lower in the PEALIFR4031 (from 1636 kW/m^2^ to 169 kW/m^2^ and from 856 kW/m^2^ to 343 kW/m^2^ respectively). In addition, the general combustion trend of the reference resembles closely the one of PEALIFR4031. Both the materials show a longer combustion time if compared with the polymer in their absence, the overall HRR intensity decreases due to the presence of the intumescent barrier, and there is a second delayed peak when this physical barrier cracks.

Wang et al. [21] have studied the effect of MH on combustion behavior of LLDPE. For 100 phr LLDPE/150 phr MH a delay of 42 s in the ignition time has been observed. This value is almost half the delay between PEAL and PEAL60MH. On the other hand, the reduction in the HRR peak intensity reported is greater than the one obtained with PEAL60MH (from 938 kW/m^2^ to 153 kW/m^2^ instead from 856 kW/m^2^ to 269 kW/m^2^). The lower reduction obtained in the present study may be attributed to the poorer quality of the recycled matrix and to the different chain morphology.

In Figure 5b is reported the comparison of the smoke production (TSR) of PEAL, PEALIFR4031 and PEAL60MH. Considering the TSR, the intumescent material behaves similarly to PEAL. In particular, smoke opacity grows almost linearly in both cases, starting from approximately 50 s. Even if for PEALIFR4031, TSR starts increasing earlier and the final value is higher (1200 m^2^/m^2^) than for PEAL (1100 m^2^/m^2^), the former reaches the final value almost 200 s later. On the other hand, the TSR starts from approximately 150 s from the beginning of the test. At the same time, also the reaching of the final value is delayed to more than 500 s. More importantly, PEAL60MH shows a small increasing of the opacity index right at the beginning. The same remains almost constant for more than 250 s and then a last increasing to the final value of approximately 212 m^2^/m^2^.

As can be seen in Figure 5b, PEAL60MH seems preferable both in terms of time of starting and finishing of TSR increasing, both in terms of speed of smoke emission.

#### 3.2.4. SEM

**Figure 6:**
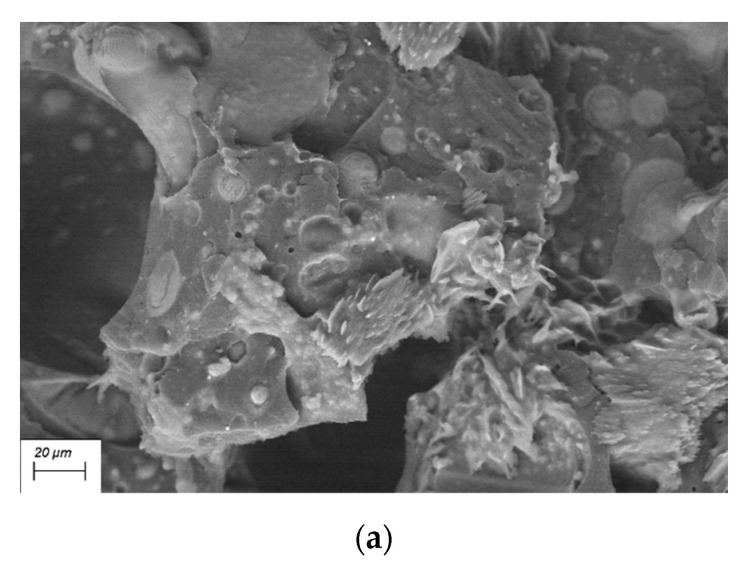

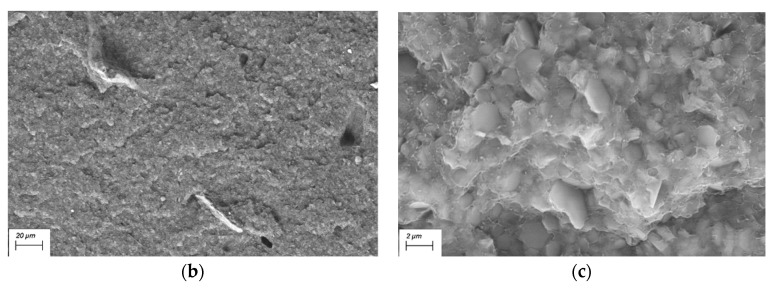
SEM image of (**a**) PEALIFR4031 1000×; (**b**) PEAL60MH 1000×; (**c**) PEAL60MH 10000×.

The section of PEALIFR4031 at 1000× is shown in Figure 6a. The material is an heterophase in which can be distinguished different agglomerates in the matrix phase. The IFR additive is arranged both in spherical and crystalline structures. This implies a poor interaction of the two IFR components and maybe one of the reasons of the poor behavior in UL-94 test. On the other hand, in Figure 6b can be appreciate the homogeneous dispersion of MH in PEAL matrix. In fact, no agglomerates can be distinguished at 1000×. Also, this can be checked in Figure 6c that shows PEAL60MH at 10000×. In particular, in the image can be seen the intimacy interaction reached between polymer and additive, that is found in the elementary hexagonal structures of about 1 µm of the main diameter.

## 4. Conclusions

In this work, the polyolefin-based material obtained from the recycling of Tetra Pak^®^ [8] has been firstly characterized on the thermostability and flammability point of view and then flame retarded with two different approaches, intumescent and inorganic additive respectively. The starting material has proven to be more stable to thermoxidative degradation if compared to LDPE. In particular, it has shown a comparable T_2%_ (294 °C for LDPE and 283 °C for PEAL) and the increase in the T_max_ from 385 °C to 421 °C. In addition, a final residue of aluminum of 14 %*w* was left. It can be concluded that the presence of aluminum flakes increases the thermoxidative stability of the matrix. The effect is even more clear considering the recycling provenance of the material, for which a worst performance should be expected. Furthermore, flammability of PEAL can be classifiable HB.

After compounding, in all cases, thermal stability in the oxidative atmosphere has improved; the weight-loss step increased up to 460–480 °C and the final residue was 21–25 %*w* for the intumescent and 38–46 %*w* for the MH-containing ones. Then, one material has been selected for each class based on the UL-94 results. In particular, PEALIFR4031, that reached V2 classification, and PEAL60MH, that achieved V0. The MH FR material has shown great enhancement also in the combustion behavior if compared with PEAL, with a decrease of 69% of the Peak HRR, of 13% of the THR and of 81% of the TSR. Also, an important increase has been observed both in the TTI (+148%) and Time of Peak HRR (+162%). On the other hand, cone calorimeter results for the intumescent material are consistent with the one of the class of FR additive chosen: if HRR is much lower than PEAL (−60%), TSR seems comparable (+9%) with the starting material and so is the TTI (−14%). It worth noting that for both the material an important increasing of the FPI has been observed, with +114% for PEALIFR4031 (FPI of 0.15) and +686% for PEAL60MH (0.55), stating that both are a valid solution to decrease the flame hazard of PEAL (0.07). In addition, the FRI parameter is in accordance, defining the flame retardancy good for the intumescent (FRI of 2.6) and excellent for the MH-containing one (10.0). In the end, the comparison in the morphology shows that a complete compounding is obtained with the inorganic additive, in fact the elementary hexagonal structures of MH have been distinguished, while IFR segregates in an heterophasic system consisting of spherical and crystalline agglomerates dispersed in the matrix.

## Figures and Tables

**Table 1:** Name and composition of the tested materials (percentages in %*w*).

Name	Composition
PEALAPP30	PEAL: 70%; APP 30%
PEALIFR3031	PEAL: 70%; IFR: 30%
PEALIFR4031	PEAL: 60%; IFR: 40%
PEAL50MH	PEAL: 50%; MH: 50%
PEAL60MH	PEAL: 40%; MH: 60%

**Table 2:** T_2%_, T_max_ and residue at 800 °C of LDPE and PEAL in inert and oxidative atmospheres.

Atmosphere	Material	T_2%_ [°C]	T_max_ [°C]	Residue at 800 °C [%]
Inert	LDPE	402	474	0
	PEAL	312	474	14
Oxidative	LDPE	294	385	0
	PEAL	283	421	14

**Table 3:** T_2%_, T_max_ and residue at 800 °C of PEALAPP30, PEALIFR3031, PEALIFR4031, PEAL50MH and PEAL60MH in oxidative atmosphere. Also, the values of PEAL are reported.

Material	T_2%_ [°C]	T_max_ [°C]	Residue at 800 °C [%]
PEAL	283	421	14
PEALAPP30	295	479	21
PEALIFR3031	213	484	23
PEALIFR4031	211	481	25
PEAL50MH	192	467	38
PEAL60MH	351	462	46

**Table 4:** Recap of cone calorimeter main results for PEAL, PEALIFR4031, PEAL60MH:TTI, Time of Peak HRR, Peak HRR, FPI, THR, TSR, FRI and Residue. In brackets, the standard deviation and the percentage variation of the parameters of the compounds compared to neat PEAL.

Material	TTI [s] (±Std. Dev.) (Δ%PEAL)	Time of Peak HRR [s] (±Std. Dev.) (Δ%PEAL)	Peak HRR [kW/m^2^] (±Std. Dev.) (Δ%PEAL)	FPI [s * m^2^/kW] (±Std. Dev.) (Δ%PEAL)	THR [MJ/m^2^] (±Std. Dev.) (Δ%PEAL)	TSR [m^2^/m^2^] (±Std. Dev.) (Δ% PEAL)	FRI (±Std. Dev.)	Residue % (±Std. Dev.) (Δ% PEAL)
PEAL	58 (±3)	183 (±13)	856 (±25)	0.07 (±0.001)	97 (±1)	1100 (±28)	-	14 (±0)
PEALIFR4031	50 (±1) (−14)	93 (±4) (−49)	343 (±18) (−60)	0.15 (±0.006) (+114)	79 (±5) (−19)	1200 (±141) (+9)	2.6 (±0.3)	33 (±1) (+136)
PEAL60MH	144 (±10) (+148)	480 (±52) (+162)	269 (±45) (−69)	0.55 (±0) (+686)	84 (±22) (−13)	212 (±41) (−81)	10.0 (±4)	48 (±0) (+242)
